# Journal of Intensive Care reviewer acknowledgement 2015

**DOI:** 10.1186/s40560-016-0132-9

**Published:** 2016-02-02

**Authors:** Satoshi Gando

**Affiliations:** Division of Acute and Critical Care Medicine, Department of Anesthesiology and Critical Care Medicine, Hokkaido University Graduate School of Medicine, ᅟ, ᅟ

## Abstract

The editors of *Journal of Intensive Care* would like to thank all our reviewers who have contributed to the journal in Volume 3 (2015).

**Mayuki Aibiki**

Japan

**Fumimasa Amaya**

Japan

**Kohkichi Andoh**

Japan

**Hideki Arimoto**

Japan

**Satoru Beppu**

Japan

**Kent Doi**

Japan

**Kentarou Dote**

Japan

**Moritoki Egi**

Japan

**Yutaka Eguchi**

Japan

**Takashi Etoh**

Japan

**Naoyuki Fujimura**

Japan

**Satoshi Fujita**

Japan

**Shigeki Fujitani**

Japan

**Shinsuke Fujiwara**

Japan

**Nobuo Fuke**

Japan

**Tatsuma Fukuda**

Japan

**Hiraku Funakoshi**

Japan

**Harumi Gomi**

Japan

**Yoshitaka Hara**

Japan

**Go Haraguchi**

Japan

**Satoru Hashimoto**

Japan

**Mineji Hayakawa**

Japan

**Yoshiro Hayashi**

Australia

**Ken-ichi Hiasa**

Japan

**Naito Hiromichi**

United States

**Mitsuru Honda**

Japan

**Patrick Honore**

Belgium

**Masami Hoshino**

Japan

**Toshiaki Iba**

Japan

**Shingo Ichiba**

Japan

**Toshiaki Ikeda**

Japan

**Yuko Ikematsu**

Japan

**Takanari Ikeyama**

Japan

**Hideaki Imanaka**

Japan

**Hideo Inaba**

Japan

**Satoki Inoue**

Japan

**Shigeaki Inoue**

Japan

**Hironori Ishihara**

Japan

**Hiroyasu Ishikura**

Japan

**Eiji Isotani**

Japan

**Shoji Ito**

Japan

**Motosh Kainuma**

Japan

**Yasuyuki Kakihana**

Japan

**Kei Kasahara**

Japan

**Shunji Kasaoka**

Japan

**Seiya Kato**

Japan

**Nobuyuki Katori**

Japan

**Kaneyuki Kawamae**

Japan

**Tatsuya Kawasaki**

Japan

**Kosaku Kinoshita**

Japan

**Hitoshi Kobata**

Japan

**Atsuko Kobayashi**

Japan

**Yutaka Kondo**

Japan

**Yoshifumi Kotake**

Japan

**Joji Kotani**

Japan

**Toru Kotani**

Japan

**Junji Kumasawa**

Japan

**Hiromitsu Kuroda**

Japan

**Yasuhiro Kuroda**

Japan

**Shigeki Kushimoto**

Japan

**Jeffrey Lipman**

Australia

**Graeme MacLaren**

Singapore

**Jun Maki**

Japan

**Ashham Mansur**

Germany

**Kazuo Maruyama**

Japan

**Yoshiki Masuda**

Japan

**Kenichi Matsuda**

Japan

**Naoyuki Matsuda**

Japan

**Hikoro Matsui**

Japan

**Francisco Maynar**

Spain

**Hiroyuki Mima**

Japan

**Chieko Mitaka**

Japan

**Toshiaki Mochizuki**

Japan

**Keita Morikane**

Japan

**Hiroshi Morimatsu**

Japan

**Hiroshi Morisaki**

Japan

**Takashi Moriya**

Japan

**Takaya Morooka**

Japan

**Susumu Nakagawa**

Japan

**Itaru Nakamura**

Japan

**Masaki Nakane**

Japan

**Atsunori Nakao**

Japan

**Hiromichi Narumiya**

Japan

**Shinichi Nishi**

Japan

**Hiroshi Nishida**

Japan

**Takeshi Nomura**

Japan

**Shin Nunomiya**

Japan

**Ryoichi Ochiai**

Japan

**Shigeto Oda**

Japan

**Masahide Ohtsuka**

Japan

**Kazufumi Okamoto**

Japan

**Satoshi Ono**

Japan

**Mutsuo Onodera**

Japan

**Yoshiro Sakaguchi**

Japan

**Nobuo Sasaki**

Japan

**Teiji Sawa**

Japan

**Atsushi Sawamura**

Japan

**Dr. Hirotaka Sawano**

Japan

**Katsuhiro Seo**

Japan

**Nobuaki Shime**

Japan

**Ayumi Shintani**

Japan

**Eiichi Suehiro**

Japan

**Gee Young Suh**

Japan

**Hidemichi Suyama**

Japan

**Takeshi Suzuki**

Japan

**Yoshio Tahara**

Japan

**Kimitaka Tajimi**

Japan

**Tetsuhiro Takei**

Japan

**Haruo Takeshita**

Japan

**Muneyuki Takeuchi**

Japan

**Naoshi Takeyama**

Japan

**Yoshiaki Terao**

Japan

**Yukichi Tokita**

Japan

**Kentaro Tokuda**

Japan

**Nelson, Nai Shun Tsoi**

Hong Kong

**Masahiko Tsuchiya**

Japan

**Ryosuke Tsuruta**

Japan

**Shigehiko Uchino**

Japan

**Osamu Umegaki**

Japan

**Akemi Utsunomiya**

Japan

**Masahiko Uzura**

Japan

**Jean-Louis Vincent**

Belgium

**Takeshi Yamamoto**

Japan

**Akira Yamashina**

Japan

**Hideto Yasuda**

Japan

**Hidekazu Yukioka**

Japan

